# Brainstem stroke preceded by transient isolated vertigo attacks

**DOI:** 10.1007/s00415-017-8610-0

**Published:** 2017-09-11

**Authors:** G. M. Halmagyi

**Affiliations:** Neurology Department, Royal Prince Alfred Hospital, Sydney, Australia

Dear Sirs,

A previously well male, a music teacher, physically active without vascular risk factors presented to our Emergency Room (ER) with an acute spontaneous vertigo attack, constant diffuse headache, and “difficulty focusing”. He was nauseated and vomited; he was unable to make any conjugate horizontal eye movements: saccadic, pursuit or vestibular. Vertical eye movements, convergence, and pupil reflexes were intact. There was no other neurological abnormality, BP was 120/80, rhythm was sinus, and cardiac evaluation was normal.

He mentioned two recent, brief, isolated spontaneous vertigo attacks. The first 3 days before; in the afternoon he was tying his kayak to the roof-rack of his car and had a sudden sensation of vertigo that resolved in a few minutes, and he did not seek medical assistance. The second occurred that night; he woke up at 4 am with severe vertigo. He had been lying on his back but was sure he had not rolled over. The vertigo was associated with nausea and vomiting. In the ER of his local hospital, his symptoms were subsiding by the time he arrived by ambulance. No clinical abnormalities were reported, a diagnosis of probable benign positional vertigo was made and he was discharged home.

**Fig. 1:**
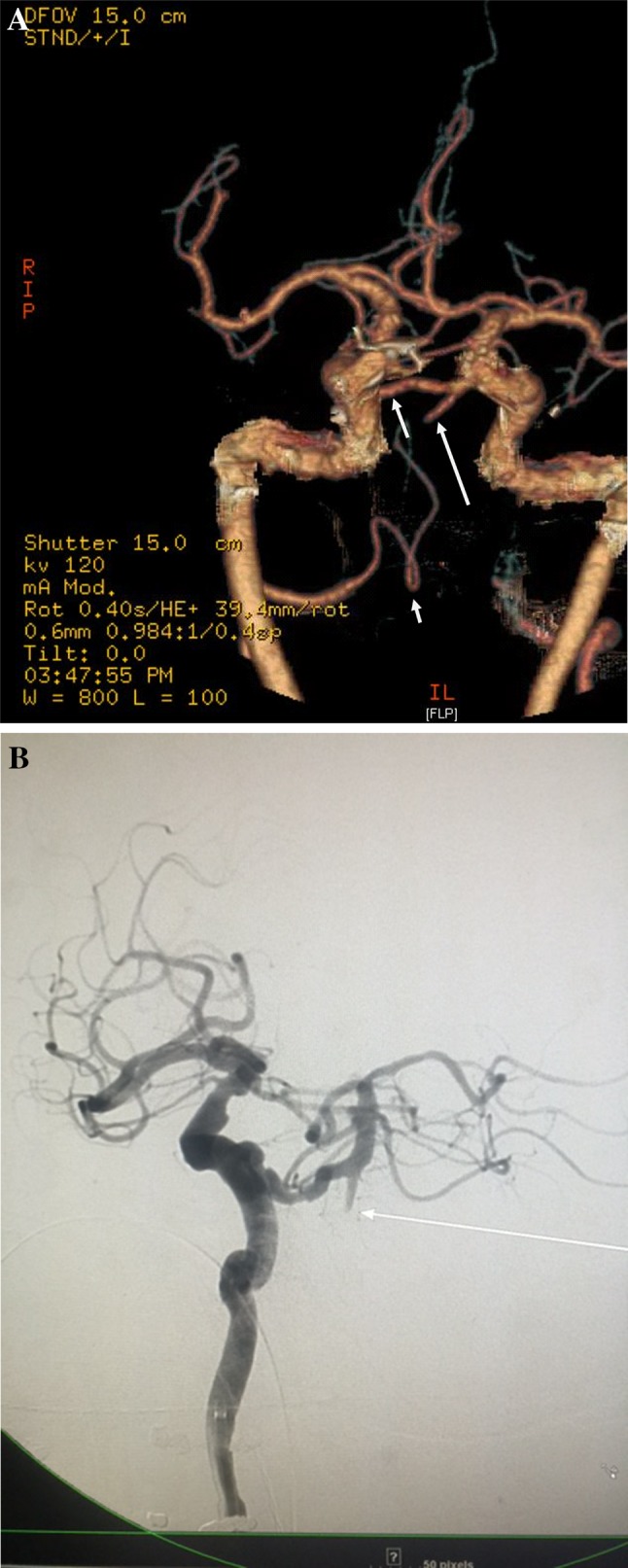
**a** CT angiogram reconstruction showing that the right vertebral artery terminates in the posterior inferior cerebellar artery (small arrow) that the distal basilar artery is supplied by a persistent trigeminal artery arising from the right internal carotid artery (midsize arrow) and that the stump of the distal left vertebral artery is just visible (large arrow). **b** Right internal carotid arteriogram showing the trigeminal artery supplying the distal basilar, arrow shows the stump of the occluded left vertebral artery

In our ER, brain CT was normal; CT angiogram (Fig. 1a) showed that the right vertebral artery terminated in the posterior inferior cerebellar artery, and that there was an abrupt cut-off of the dominant left vertebral artery at the level of the lower pons. Both superior cerebellar arteries and both posterior cerebral arteries opacified normally as did the distal basilar artery, from a persistent trigeminal artery arising from the right internal carotid artery.

**Fig. 2:**
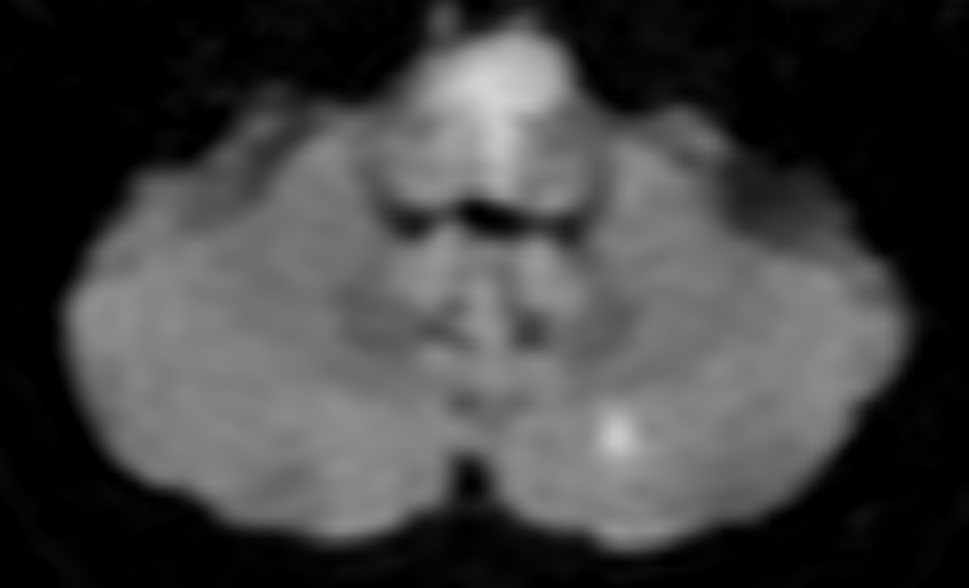
DWI MRI showing the typical heart-shaped appearance of bilateral medial medullary infarcts. There is also a small infarct in the left posterior inferior cerebellar artery territory

He was admitted to our acute stroke unit and given aspirin. After 24 h he suddenly developed dysarthria, dysphagia, a right hemiparesis and hemianesthesia. CT angiogram now showed that the left vertebral artery thrombosis had extended into the proximal basilar artery. MRI (Fig. 2) showed acute bilateral infarcts of the ventromedial medulla, dorsal mid-pons and left cerebellar hemisphere. A heparin infusion was started. He needed to be intubated. With a view to thrombectomy a catheter cerebral arteriogram was done which confirmed the CT angiogram findings; however, the left vertebral was too tortuous and the procedure was abandoned. The patient was anticoagulated for 2 weeks and aspirin was continued. He was transferred for inpatient rehabilitation 2 months later. A year and a half later, his hemiplegia has almost resolved but he still has a horizontal gaze paresis.

It is still a valid adage that isolated vertigo attacks, spontaneous or positional, are generally due to benign peripheral vestibular disease, and are rarely due to vertebrobasilar ischaemia [1]. Rarely, but not never [2, 3]. Prospectively less than 5% of patients with vertigo seen in ER and discharged or admitted to hospital, have a stroke in the next 3 years [4–6]. Retrospectively about 20% of patients who have had a brainstem stroke report having had isolated vertigo attacks, usually only for a few days before the stroke [7, 8]. Therefore, it is rare for patients to have isolated vertigo attacks of cerebrovascular origin for long enough to be seen by appointment; they will usually have a stroke long before then. So the problem our patient had is not exceptional and has important management implications.

I wondered, what I, a neuro-otologist, could/would/should have done if I had been called to ER to see this patient after his second, self-limiting vertigo attack. I too would have noted that he had fully recovered from his severe, spontaneous vertigo attack lasting about an hour, associated with nausea and vomiting but not with headache, hearing problems or any other neurologic symptoms. He had no vascular risk factors, and now no neurologic or vestibular signs, specifically no nystagmus, a negative Dix–Hallpike test and a negative head impulse test.

Would I also have not considered that the vertigo was more likely to have been due to BPV or Meniere’s disease than to a transient ischemic attack or small stroke? Hopefully not. I would, or at least should, have noted that while the brief vertigo attack he had looking up to the roof-rack of his car might have been BPV, the attack that then woke him up during that night did not sound like BPV—he was sure he had not rolled over and it was still present when he then sat up. It also did not sound like Meniere’s—the first vertigo attack is rarely so severe and his hearing was, at least subjectively, unaffected. It did not sound like migraine—he never had headaches. So what would that have left as a possible diagnosis? Really, only vertebrobasilar ischaemia. Or as Sherlock Holmes supposedly said: “When you have eliminated the impossible, whatever remains, however improbable, must be the truth”. So what should/could have I done, immediately? MRI/MRA would have shown his congenital posterior circulation abnormalities [9–11], probably a dangerous stenotic plaque in his dominant left vertebral artery and perhaps a small asymptomatic acute cerebellar infarct [12]. I certainly would have given him aspirin then [13], and also considered urgent stenting of the stenotic left vertebral artery [14, 15]. Would that have made much difference? Hard to know. Nonetheless, this case emphasizes that a first-ever, isolated, acute vertigo attack, even if transient, like all first-ever attacks in neurology, is potentially dangerous, until proven otherwise, and needs to be taken seriously [16]. Details of this patient’s selective horizontal gaze palsy have been published [17].
